# Patterns of radioiodine use for differentiated thyroid carcinoma in Brazil: insights and a call for action from a 20-year database

**DOI:** 10.20945/2359-3997000000302

**Published:** 2020-10-21

**Authors:** Wallace Klein Schwengber, Laís Marques Mota, Carla Fernanda Nava, João Antônio Paim Rodrigues, André B. Zanella, Ricardo De Souza Kuchenbecker, Rafael Selbach Scheffel, Ana Luiza Maia, Jose Miguel Dora

**Affiliations:** 1 Universidade Federal do Rio Grande do Sul Faculdade de Medicina Hospital de Clínicas de Porto Alegre Porto Alegre RS Brasil Unidade de Tireoide, Hospital de Clínicas de Porto Alegre, Faculdade de Medicina, Universidade Federal do Rio Grande do Sul, Porto Alegre, RS, Brasil; 2 Hospital de Clínicas de Porto Alegre Porto Alegre RS Brasil Coordenação Administrativa, Hospital de Clínicas de Porto Alegre, Porto Alegre, RS, Brasil; 3 Universidade Federal do Rio Grande do Sul Faculdade de Medicina Departamento de Epidemiologia Porto Alegre RS Brasil Departamento de Epidemiologia, Faculdade de Medicina, Universidade Federal do Rio Grande do Sul, Porto Alegre, RS, Brasil; 4 Universidade Federal do Rio Grande do Sul Instituto de Ciências Básicas da Saúde Departamento de Farmacologia Porto Alegre RS Brasil Departamento de Farmacologia, Instituto de Ciências Básicas da Saúde, Universidade Federal do Rio Grande do Sul, Porto Alegre, RS, Brasil; 5 Hospital de Clínicas de Porto Alegre Porto Alegre RS Brasil Serviço de Medicina Interna, Hospital de Clínicas de Porto Alegre, Porto Alegre, RS, Brasil; 6 Universidade Federal do Rio Grande do Sul Faculdade de Medicina Departamento de Medicina Interna Porto Alegre RS Brasil Departamento de Medicina Interna, Faculdade de Medicina, Universidade Federal do Rio Grande do Sul, Porto Alegre, RS, Brasil

**Keywords:** Differentiated thyroid carcinoma, radioiodine, treatment

## Abstract

**Objective:**

This study aimed to explore the patterns of radioactive iodine (RAI) use for differentiated thyroid cancer (DTC) in Brazil over the past 20 years.

**Materials and methods:**

A retrospective analysis of the DTC-related RAI prescriptions, from 2000 to 2018, retrieved from the Department of Informatics of the Unified Health System (Datasus) and National Supplementary Health Agency (ANS) database was performed. RAI activities prescriptions were re-classified as low (30-50 mCi), intermediate (100 mCi), or high activities (>100 mCi).

**Results:**

The number of DTC-related RAI prescriptions increased from 0.45 to 2.28/100,000 inhabitants from 2000 to 2015, declining onwards, closing 2018 at 1.87/100,000. In 2018, population-adjusted RAI prescriptions by state ranged from 0.07 to 4.74/100,000 inhabitants. Regarding RAI activities, in the 2000 to 2008 period, the proportion of high-activities among all RAI prescriptions increased from 51.2% to 74.1%. From 2009 onwards, there was a progressive reduction in high-activity prescriptions in the country, closing 2018 at 50.1%. In 2018, the practice of requesting high-activities varied from 16% to 82% between Brazilian states. Interestingly, variability of RAI use do not seem to be related to RAI referral center volume nor state socio-economic indicators.

**Conclusion:**

In recent years, there has been a trend towards the lower prescription of RAI, and a reduction of high-activity RAI prescriptions for DTC in Brazil. Also, significative inter-state and inter-institutional variability on RAI use was documented. These results suggest that actions to advance DTC healthcare quality surveillance should be prioritized.

## INTRODUCTION

Differentiated thyroid carcinoma (DTC) comprising papillary and follicular carcinoma account for the majority of thyroid malignancies, is responsible for almost 3.1% of all new diagnosis of cancers in 2018 worldwide ( 1 ). The increasing incidence of thyroid cancer, previously observed mainly in high-income countries, is also a real phenomenon in low- and middle-income countries ( 2 ). In Brazil, the statistics provided by the National Cancer Institute (INCA) for 2018 estimate an annual incidence of 9,610 new thyroid cancer cases, with an estimated incidence of 1.49 and 7.57 per 100,000 habitants/year for men and women, respectively ( 3 ).

Management of DTC is under continuous review and has changed substantially in recent years. Given a significant amount of new information in the field, physicians rely on Guidelines to embase care decisions. Guidelines serve as an evidence-based repository of knowledge, qualifying decisions and minimizing undesirable clinical variability, thus contributing to increased healthcare value. The DTC Guidelines of the American Thyroid Association (ATA) are under continuous review, and the last version has been released in 2015 ( 4 ). Nonetheless, despite the efforts to increase the uniformity of care, considerable variability of DTC management regarding radioiodine (RAI) use is well documented ( 5 , 6 ).

RAI utilization for DTC treatment dates from the 1940s ( 7 , 8 ) and as an adjuvant treatment can be prescribed in different activities, ranging from as low as 30 mCi to over 250 mCi. The goals of RAI therapy should be based on individual patients risk, and can be for three purposes: RAI remnant ablation facilitating the early detection of recurrent disease; RAI adjuvant therapy minimizing the risk of residual disease and RAI therapy for persistent and/or metastatic disease. Initially, RAI was recommended to all patients with DTC. However, more recent evidence, that applies especially to low- and intermediate-risk DTC suggests that the effectiveness of remnant ablation with 30 mCi was similar to treatment with 100 mCi ( 9 - 11 ). Additionally, two recent meta-analyses bring to light the risk of permanent non-malignant adverse effects on salivary, lacrimal, and gonadal dysfunction ( 12 ), as well as an increased risk of second malignancy related to cumulative RAI dosing ( 13 ). Additionally, the use of lower activities of RAI, instead of high activities, contribute to lowering health care system costs and environmental impact from radiation. Aligned with this evidence and given the lack of superiority of high activities of RAI for most DTC patients, RAI indications for DTC are being narrowed towards the use of lower activities in the last decades.

Few studies addressed country-wide patterns of DTC RAI treatment ( 14 - 16 ). This study aims to explore the temporal patterns of RAI use for diagnosed cases of DTC over the past 20 years in Brazil and its states.

## MATERIALS AND METHODS

The Brazilian Unified Health System (SUS), a public-private mix, has three parts: the public subsector, the private subsector and the private health insurance subsector. Most of the Brazilian health services are provided by the public subsector, where services are provided and financed by the government at the federal, state and municipal levels to nearly 210 million inhabitants ( 17 ). Additionally, nearly 25% of the Brazilian population count on private health insurance healthcare, regulated by the National Agency for Supplementary Health (ANS). Hence, a retrospective study was performed in January and February 2020, in which the primary sources of information were the Department of Informatics of the SUS (Datasus, http://datasus.saude.gov.br ) ( 18 ) and the ANS databases ( http://ans.gov.br ) ( 19 ).

Data were collected from 2000 to 2018, following Datasus codes for thyroid cancer management-related procedures ( Supplemental Table 1 ). In the Datasus Outpatient Information System, the number of oncologic thyroidectomies and RAI medical prescriptions for different activities (30, 50, 100, 150, 200 and 250 mCi) are registered according to the state of origin, region (South, Southeast, Northeast, North, and Midwest) and for the entire country.

Data on RAI prescriptions for DTC in the private health insurance subsector was gathered from the ANS website by the state of origin, region and for the entire country ( Supplemental Table 1 ). Data on RAI prescriptions were available in Datasus from 2000 to 2018, while ANS data were available for years 2015, 2016, 2017 and 2018.

The Brazilian National Cancer Institute ( https://www.inca.gov.br/ ) provides annual estimates on newly diagnosed thyroid cancer cases for each state, applying the method proposed by Black and cols. ( 20 ). This information was employed to standardize the number of RAI prescriptions for new DTC cases, for each state, for the year of 2018.

Population estimates from 2000 to 2018 was obtained from the Brazilian Institute of Geography and Statistics (IBGE, https://ibge.gov.br/ ), stratified annually by states, by regions, and for the entire country. The data were analysed with the total RAI medical activity prescriptions, to describe RAI prescriptions standardized to the population of the area for a given year.

Considering the complementary characteristics of public (SUS) and private (ANS) assisted cases, we then calculated DTC RAI prescriptions standardized per 100,000 inhabitants, dividing all DTC RAI prescriptions (SUS and ANS) by the estimated population of the given area and period. Then RAI prescriptions, adjusted for cancer-related thyroidectomies, were calculated by dividing public subsector SUS DTC RAI prescriptions by SUS oncologic thyroidectomies in the area for the given period. Similarly, public and private subsectors RAI prescriptions, adjusted for the INCA DTC new cases estimates, were calculated by dividing all public and private subsectors DTC RAI prescriptions (SUS and ANS) by the INCA DTC new cases estimates for the area for the given period.

We organized DTC RAI prescriptions throughout the 2000-2018 period and re-classified them according to the following levels: low (30 and 50 mCi), intermediate (100 mCi), or high activities (>100 mCi). The proportion of high RAI prescriptions activities was calculated dividing the number of SUS DTC RAI prescriptions with more than 100 mCi by all SUS DTC RAI prescriptions (high, intermediate and low) for the area for the given period. Of note, the ANS database does not provide RAI activity information. Thus, the analysis of RAI activity patterns is based only on the Datasus database.

To explore the potential influence of socioeconomic factors on the patterns of RAI use across states in Brazil, we also gathered the most recent socioeconomic indicators data available, as follows: Human Development Index (HDI) from Brazil United Nations Development Programme (UNDP) for the year of 2017, Gross Domestic Product (GDP) per capita from Regional Account System: Brazil 2017 ( *Sistema de Contas Regionais: Brasil* 2017), Gini Index from National Household Sample Survey ( *Pesquisa Nacional por Amostra a Domicílios* – PNAD) for the year of 2013 and ANS coverage by state from the ANS website. The HDI uses distinct indicators to display a measure of average achievement in key dimensions of human development: a long and healthy life, being knowledgeable and have a decent standard of living. GDP is a metric that breaks down a region economic output per person and is calculated by dividing the GDP of a region by its population, aiming to assess wealth and prosperity. The Gini index measures the extent to which the distribution of income among individuals or households within an economy deviates from a perfectly equal distribution. Finally, the ANS coverage by state represents an indicator of the extent of the private health insurance healthcare availability for the population of each state.

The project was approved by the ethics committee of *Hospital de Clínicas de Porto Alegre* (CAAE 29670919.9.0000.5327/GPPG 2019-0764), and all authors vouch for the accuracy of the data and analyses.

## RESULTS

### Number of DTC RAI prescriptions in Brazil

**Figure 1:**
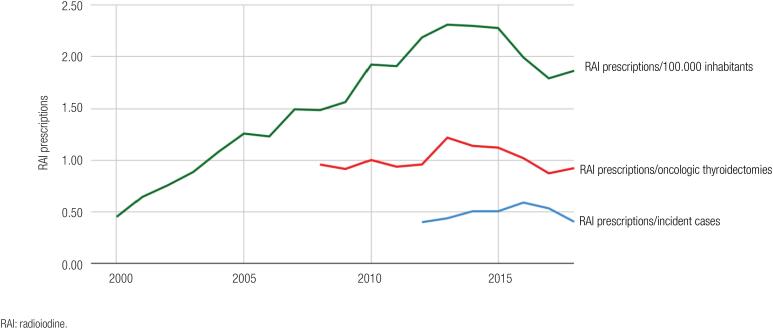
Radioiodine (RAI) prescriptions rates adjusted for population estimates, for oncologic thyroidectomies and DTC new cases in Brazil, from 2000 to 2018.

From 2000 to 2015, the population-adjusted DTC RAI prescriptions in Brazil increased from 0.45 to 2.28 per 100,000 inhabitants ( Figure 1 ). Since 2015, the number of DTC RAI prescriptions showed a declining tendency in Brazil, closing 2018 at 1.87 per 100,000 inhabitants.

Data Sources: Datasus and IBGE.

When analysing RAI prescriptions adjusted for the number of cancer-related thyroidectomies in Brazil, we noticed a double-pattern tendency: firstly, there was an increase from 0.96 to 1.22 SUS DTC RAI prescriptions per cancer-related thyroidectomy between 2008 and 2013. Since then, the number of SUS DTC RAI prescriptions adjusted for cancer-related thyroidectomies declined, reaching 0.94 in 2018 ( Figure 1 ).

We also assessed the number of RAI prescriptions adjusted for the INCA DTC new cases estimate from 2012 to 2018 ( Figure 1 ). The estimated number of RAI prescriptions per new DTC case was 0.40 in 2012, reaching 0.59 in 2016 and returned to 0.40 in 2018.

### RAI activities in Brazil

**Figure 2:**
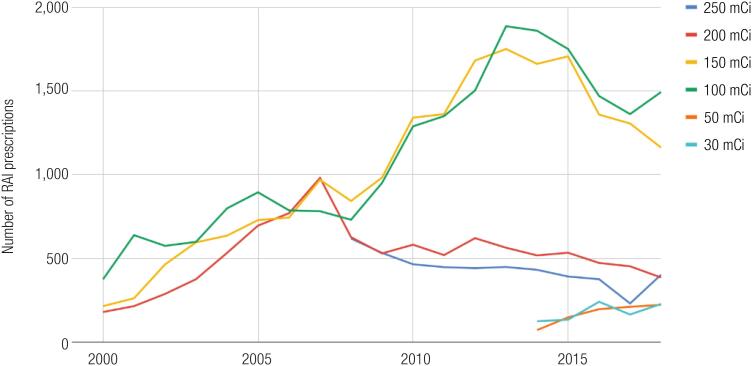
Patterns of radioiodine use for DTC in Brazil, and stratified by RAI activity, from 2000 to 2018.

We then analysed the proportions of DTC RAI prescriptions with high (>100 mCi), intermediate (100 mCi), and low (30-50 mCi) activity, from 2000 to 2018 ( Figure 2 ). Again, we observed a double pattern: in the 2000-2008 period, the proportion of high activities among all RAI prescriptions increased from 51.2% to 74.1%. After 2008, the proportion of high activities decreased, reaching 50.1% in 2018. Low activities of RAI for DTC, which are registered by Datasus since 2014, increased from 4.2% in 2014 to 11.5% in 2018.

Data Source: Datasus.

### Heterogeneity of Brazilian states

In the years of 2017 and 2018, we identified respectively, 4,758 and 5,074 RAI prescriptions for DTC in Brazil. The SUS RAI prescriptions represented, respectively 3,722 (78%) and 3,890 (77%) of all RAI prescriptions for DTC for 2017 and 2018.

**Figure 3:**
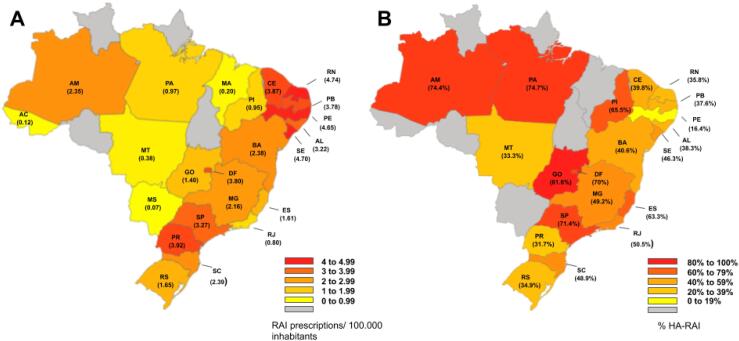
The number of RAI prescriptions per 100.000 inhabitants and per cent of high RAI activities, in Brazil by states for the year 2018.

To explore the variability in DTC RAI treatment among the Brazilian states, we show a 2018 snapshot of the RAI adjusted prescriptions for 100.000 inhabitants and the proportion of high RAI activities ( Figure 3 ). This data demonstrates that the practice of ordering high RAI activities varied from 16% in Pernambuco to 82% in Goiás and that RAI prescriptions adjusted to 100,000 inhabitants, varied from 0.07 in Mato Grosso do Sul to 4.74 in Rio Grande do Norte.

Data Sources: Datasus, ANS and IBGE.

We then explored whether socioeconomic indicators or RAI referral center volume contribute to the variability of RAI practices identified. We found no correlation between the number of RAI prescriptions/100,000 inhabitants and %HA-RAI with four socioeconomic indicators (HDI, GDP, Gini index and ANS coverage) ( Table 1 ).

**Table 1:** Correlation between the patterns of use of radioiodine and socioeconomic indicators for Brazilian states

	Human Development Index (HDI) (2017)	Gross Domestic Product (GDP) per capita (2017)	Gini Index (2013)	ANS coverage (2018)
Per cent high RAI activities (2018)	0.153 p = 0.507	0.167 p = 0.469	0.009 p = 0.970	0.140 p = 0.544
RAI prescriptions/100,000 inhabitants (2018)	0.115 p = 0.569	0.109 p = 0.587	0.063 p = 0.754	0.302 p = 0.126

RAI: radioiodine.

Data Sources: Datasus, ANS, IBGE, UNDP, RAS and PNAD.

Considering that referral center volume could explain part of the variability of health care, we looked at patterns of RAI use according to RAI referral center volume. In 2018, according to the Datasus database, 53 institutions administered RAI activities ≥100 mCi. The state of São Paulo accounted for 15/53 (28%) of the number of RAI institutions, and together with other four Brazilian states (MG, PR, RS and RJ) registered 35/53 (66%) of RAI institutions. Three states (BA, PB and PE) had two RAI institutions registered and other 12 states one RAI institution (AL, AM, CE, DF, ES, GO, MT, PA, PI, RN, SC and SE). Seven states had no RAI institutions (AC, AP, MA, MS, RO, RR and TO).

We then looked at the number of RAI prescriptions per institution ( Supplemental Table 2 ). In 2018, 11 (21%) institutions prescribed more than 100 inpatient RAI activities (High RAI volume institutions), another 11 (21%) between 50 and 100 inpatient RAI activities (Intermediate RAI volume institutions), and 31 (58%) less than 50 inpatient RAI activities (Low RAI volume institutions). We looked at the proportion of inpatient high RAI activities prescribed according to institution volume: high volume institutions prescribed 1079/2020 (53%) high RAI activities, intermediate volume institutions 559/848 (66%) high RAI activities, and low volume institutions 310/573 (54%) high RAI activities. Additionally, there was no correlation between the number of inpatient RAI activities prescribed by institution and the proportion of high RAI activities (R = 0.006). Taking together these findings do not support that RAI practices for DTC therapy accompany institutional RAI prescriptions volume.

## DISCUSSION

Here we demonstrated that the use of RAI and high RAI activities for DTC in Brazil seems to be decreasing. Nonetheless, in contrast with current international clinical guidelines, the proportion of high RAI activities is still surprisingly high. Additionally, we documented a significant variability of coverage and patterns of care regarding RAI use for DTC between different Brazilian institutions and states. We also believe that data on DTC treatment should be better organized to make it readily available and allow its users to monitor and evaluate treatments and outcomes. Better access will facilitate a reduction in treatment heterogeneity and improve access to health care across Brazil.

In recent years there is a growing consensus that many DTC patients do not benefit from RAI treatment, and that a considerable portion of DTC patients can be treated with low RAI activities, reducing treatment-related damage. Thus, the observed reduction in DTC RAI prescriptions in recent years shows that clinical practice has this trend indeed. Another aspect to highlight is that DTC RAI prescriptions and high RAI activities seem to decline and follow recent changes in guideline recommendations despite the evolving healthcare coverage in Brazil. Nonetheless, the proportion of high RAI activities still represents more than 50% of DTC RAI prescriptions across the country. Given the lack of benefit of high RAI activities to most DTC cases and the well-known adverse effects due to unnecessary increased exposure to radiation, the practice of ordering high RAI activities for DTC is worrisome. The unjustified use of high RAI activities increase the risk of radiation-induced benign and malignant complications and pose an economic burden to the health system and the environment ( 21 ). We understand that RAI therapy for persistent and/or metastatic disease may be eligible to RAI activities higher than 100 mCi. However, most DTC cases are restricted to the thyroid gland (∼70%), around 30% presents cervical lymph node metastasis and only ∼5% distant metastasis ( 4 ). Thus, one should note that the indication of high RAI activities may apply to a small subgroup of DTC patients. Notwithstanding, still for this subgroup of advanced DTC, the optimal therapeutic activity remains uncertain and controversial.

Another aspect to consider is the disparities in Brazilian health care coverage. Some Brazilian states still do not have access to RAI therapy, so adequate DTC adjuvant treatment is not provided for a substantial portion of the affected cases. This problem is not restricted to the public subsector because RAI prescriptions are not registered both either SUS or ANS healthcare beneficiaries in some states. In this matter, our study underscores not only overuse of treatment strategies in some states, but also underuse of RAI for DTC all over Brazil. Within this context, our data documents considerable DTC variability of health care among Brazilian states. Several factors may explain these findings. First, the low quality of evidence on DTC RAI treatment indicates clinical practice variability, a well-described phenomenon documented in previous studies from other countries ( 5 , 6 , 22 ). Second, economic and regional issues are of particular interest in Brazil, since the heterogeneous access to diagnostic and treatment delineate decision pathways with different tradeoffs according to the particular contexts. Interestingly, however, DTC RAI variability of health care documented do not seem to be explained by socioeconomic indicators nor RAI center volume, indicating that other variables must come into play.

We should also comment on the limitations of this study. The Datasus and ANS databases, the best known national data available, are comprised of aggregated ecological data. Thus, as these systems were not designed as cancer registries, they are prone to bias. Notwithstanding, given that both Datasus and ANS systems are based on billing information, they are audited by competent authorities, what contributes for a minimal curadorie of the data. This fact is also a call for action to work towards a national consolidated DTC database, that encompasses individual patient and tumor characteristics, as well as diagnostic and therapeutic resources used, along with clinical outcomes. Also, the proposed metrics on RAI use are new, a fact that limits benchmarking with other healthcare scenarios and countries. The studied estimates relay on diagnosed cancer cases, and therefore may be prone to some level of detection bias. Taken together, these factors may result in less accurate estimates over the studied period. Thus, it would be interesting if our findings could be complemented by multi-institutional health record review based-study.

In summary, the observed estimates of RAI over- and underuse, the practice of ordering high activities and the significant variability of provided health care among states determine the predominant pattern of RAI use for DTC in Brazil. Also, population-adjusted DTC RAI prescriptions, DTC RAI prescriptions adjusted to new cases or cancer-related thyroidectomies, and percentage of high activity RAI prescriptions emerge as potential valuable metrics to compare, monitor, and evaluate DTC RAI patterns of treatment. Our findings are descriptive but may serve as a call for action, highlighting significant opportunities to improve DTC care in Brazil, and suggesting that advanced surveillance of DTC healthcare quality and improvement of the national cancer registry system should be prioritized.

## Supplemental Table 1 Unique Health System and the National Supplementary Health Agency Thyroid Cancer Radioiodine Procedures in Brazil.

**Table:**

Procedure	Datasus Code(s)	Period
RAI 30 mCi	03.04.09.005-0	From Feb/2014 to present
RAI 50 mCi	03.04.09.006-9	From Feb/2014 to present
RAI 100 mCi	03.04.09.002-6	From Jan/2008 to present
	85.300.888 and 85.500.887	From Nov/1999 to Dec/2007
RAI 150 mCi	03.04.09.001-8	From Jan/2008 to present
	85.300.900 and 85.500.909	From Nov/1999 to Dec/2007
RAI 200 mCi	03.04.09.003-4	From Jan/2008 to present
	85.300.926 and 85.500.925	From Nov/1999 to Dec/2007
RAI 250 mCi	03.04.09.004-2	From Jul/2008 to present
	03.03.12.001-0	From Jan/2008 to Jun/2008
TT in oncology	0416030270	From 2013 to present
	0416030130	From Ago/2008 to 2013
TT w/lymph node resection	0416030122	From Jan/2008 to 2013
	0402010051	From Jan/2008 to present
Transsternal thyroid tumor resection in oncology	0416030360	From 2013 to present
	0416030050	From Jan/2008 to 2013
**Procedure**	**ANS Code**	**Period**
Treatment of thyroid cancer (nuclear therapy)	40710041	From 2015 to 2018

Data source: Tabnet/Datasus ( tabnet.datasus.gov.br ) and ANS ( http://www.ans.gov.br/perfil-do-setor/dados-e-indicadores-do-setor/d-tiss ).

RAI: radioiodine treatment. TT: total thyroidectomy

## Supplemental Table 2 Number of inpatient RAI activities administered by institution for the year of 2018 in Brazil

**Table:**

Institution	State	Inpatient RAI Activity Administered (mCi)				
		100	150	200	250	All
2688689 SANTA CASA DE SÃO PAULO HOSPITAL CENTRAL SÃO PAULO	SP				317	317
2723220 HOSPITAL HAROLDO JUAÇABA	CE	145	112	10	1	268
0003786 HOSPITAL ARISTIDES MALTEZ	BA	124	71	49	10	254
2090236 FUNDAÇÃO PIO XII BARRETOS	SP	82	115	39	7	243
2409194 HOSPITAL DR. LUIZ ANTONIO	RN	106	46	13		165
6123740 INSTITUTO DO CÂNCER DO ESTADO DE SÃO PAULO	SP	83	42	24		149
0000434 IMIP	PE	139	1	4		144
2200457 ASSOCIAÇÃO MARIO PENNA	MG	61	53	18	1	133
2302969 ICSC	SC	68	50	10	5	133
2707497 DIAGNOSTICA	PB	67	30	8	2	107
2007037 SANTA CASA DE MISERICÓRDIA DE MACEIÓ	AL	66	35	3	3	107
0015644 HOSPITAL ERASTO GAERTNER	PR	49	39	9		97
2077590 IBCC	SP		91			91
2012677 FUNDAÇÃO CECON	AM	23	66	1		90
2506815 HOSPITAL DE CÂNCER	GO	16	23	41	7	87
2577623 HCL HOSPITAL DO CÂNCER DE LONDRINA	PR	48	32	6		86
0003417 FUNDAÇÃO CLIMEDI	SE	44	36	2		82
0000396 HOSPITAL DAS CLÍNICAS	PE	25	25	21	6	77
2334321 HOSPITAL OPHIR LOYOLA	PA	19	31	15	10	75
2079798 HOSPITAL DAS CLÍNICAS DA UNICAMP DE CAMPINAS	SP	19	30	4	7	60
2083086 HOSPITAL AMARAL CARVALHO JAU	SP	19	26	7		52
2237571 HOSPITAL NOSSA SENHORA DA CONCEIÇÃO S.A.	RS	27	23	1		51
2195453 HOSPITAL DO CÂNCER DE MURIAÉ	MG	21	20	6	2	49
2273454 MS INCA HOSPITAL DO CÂNCER I	RJ	14	17	12	3	46
2237601 HOSPITAL DE CLÍNICAS	RS	42	1			43
2080532 SANTA CASA HOSP. DR. ARISTÓTELES OLIVEIRA MARTINS PRES. PRUDENTE	SP			36	3	39
2077396 HOSPITAL DE BASE DE SÃO JOSÉ DO RIO PRETO	SP	25	8	2		35
0011738 HOSPITAL SANTA RITA DE CASSIA VITÓRIA	ES	11	14	2	3	30
2737434 CEONC	PR	29		1		30
2726998 HOSPITAL SÃO MARCOS	PI	10	11	3	5	29
0013633 HOSPITAL ANGELINA CARON	PR	12	13	4		29
2165058 HOSPITAL DOUTOR HÉLIO ANGOTTI	MG	11	11	3		25
2740338 HOSPITAL DO CÂNCER DE CASCAVEL UOPECCAN	PR	16	6	3		25
2269988 MS HSE HOSPITAL DOS SERVIDORES DO ESTADO	RJ	11	10	1		22
2748223 HOSPITAL DAS CLÍNICAS DA FACULDADE DE MEDICINA DE BOTUCATU	SP	3	12	5	2	22
2077531 A.C.CAMARGO CANCER CENTER	SP	1	14		3	18
2261057 HOSPITAL DE CARIDADE DE IJUÍ	RS	4	8	5		17
0003808 HOSPITAL SÃO RAFAEL	BA	7	5	1	2	15
2153106 ONCOLÓGICO	MG	7	4	2		13
2078775 SANTA CASA DE ARAÇATUBA HOSPITAL SAGRADO CORAÇÃO DE JESUS	SP		5	8		13
2082187 HOSPITAL DAS CLÍNICAS FAEPA RIBEIRÃO PRETO	SP	12		1		13
0010456 HOSPITAL DE BASE DO DISTRITO FEDERAL	DF	3	6	1		10
2707918 FUNDAÇÃO HOSPITALAR SANTA TEREZINHA DE ERECHIM	RS	7	1	1		9
2278855 HOSPITAL SÃO JOSE DO AVAÍ	RJ	6		2		8
2705982 SANTA CASA DE FRANCA	SP		6			6
2237253 IRMANDADE DA SANTA CASA DE MISERICÓRDIA DE PORTO ALEGRE	RS	2	4			6
2129469 SANTA CASA DE POÇOS DE CALDAS	MG		2		3	5
2280167 UFRJ HOSPITAL UNIVERSITÁRIO CLEMENTINO FRAGA FILHO	RJ	3	1			4
2270803 SES RJ I INST. EST. DIABET. ENDOCRINOLOGIA IEDE	RJ		3			3
2089327 HOSPITAL PADRE ALBINO CATANDUVA	SP	3				3
2534444 HOSPITAL DE CÂNCER DE MATO GROSSO	MT	2		1		3
3112691 DIAGSON	PB	1	1			2
2798298 SANTA CASA DE MISERICÓRDIA DE SÃO JOSÉ DO RIO PRETO	SP		1			1
All institutions	Brazil	1493	1161	385	402	3441

Data Sources: Datasus.
